# The Pharmacopeia—As Seen by One General Practitioner

**Published:** 1907-05

**Authors:** J. Leverett

**Affiliations:** Yonkers, N. Y.


For Texas Medical Journal.

### The Pharmacopeia—As Seen by One General Practitioner.

BY J. LEVERETT, A. B., M. D., YONKERS, N. Y.

Why is not the U. S. Pharmacopeia more popular with the physicians? Why do not a larger proportion of prescriptions call for pharmacopeial drugs? Is there any good reason on the part of the physician why he should use the pharmacopeia more? Is there any good reason for its lack of popularity?

When a student graduates into a full-fledged physician he has passed an examination in materia medica, and that materia medica is composed principally, if not entirely, of pharmacopeial drugs.

The first few years in practice he is gaining experience, has time to study his cases pretty thoroughly, and is prejudiced in favor of the pharmacopeia. He gets familiar with certain drugs which in this combination or that appear to influence certain conditions for good. Certain others which he has been taught to depend upon have failed him and a proprietary has seemed to turn the trick.

At the end of eight or ten years he has quite a pharmacopeia of his own, limited, to be sure, but composed of drugs which he feels that he can depend upon, some official and some non-official.

One of these is, perhaps, tincture digitalis, or tincture belladonna, or tincture aconite. He has gotten way beyond the textbook in these drugs and his dosage does not depend on a memorized strength, but on the effect which he has been accustomed to get from a certain quantity.

Just now a revision of the pharmacopeia becomes authoritative, and he wonders why his prescriptions containing these tinctures do not have the effect he has learned to expect. He now learns that the strength of these tinctures has been materially decreased. He makes a mental note of this and tries to arrange his prescriptions accordingly, but he has come to think of tincture digitalis as an entity with certain effects and not as a certain percentage of a crude drug which in its crude form has never entered into his experience, and therefore he finds himself more or less at sea when writing prescriptions containing that tincture. What wonder then if he falls back on a proprietary which he has until now used but frequently but which he feels it is to the interest of the manufacturer to keep at a standard strength and which therefore he can depend upon. But for the next changes in the pharmacopeia we are not to wait ten years. The druggists are clamoring for an annual revision to be issued in a supplement. But, you ask, what have the druggists to do with it? Is not the pharmacopeia in the hands of the physicians?

For answer let us look at the composition of the revision committee. Fifteen druggists and eleven physicians, and of these physicians more of them theorists than practical men.

Am I trying to block the wheels of progress? Not at all. A revision every year if you choose, but make it in the interests of the physician. Let the changes be the adding of new preparations, and the cutting out of obsolete ones, but hands off the old standbys.

But why should the physician make a fetich of the pharmacopeia? We can see why the druggist should be interested and why he should wish that prescriptions be limited to a certain list of drugs, but since when have the physicians been in leading strings to be dictated to by any body of men whether they are called a committee on revision or a committee on new and non-official preparations?

The physician's business is to carry on the battle against disease and to use the means, whether official or not, which will give the victory to his patient. It is the business of the pharmacist to see that the drugs furnished on prescriptions are what the physician expected his patient to receive, and it is not his province to try to tie the physician down to a certain set of drugs. The individual physician has to shoulder the responsibility in each case and his hands should be perfectly free.

---

MARRIED at Houston, Texas, April 13th, Dr. Frank R. Ross to Mary, daughter of Dr. D. F. Stuart, all of Houston.

